# Efficacy and Safety of Hydroxyurea as Adjuvant Therapy in Pediatric Patients of Transfusion-Dependent Beta-Thalassemia Major at Zhob, Balochistan

**DOI:** 10.7759/cureus.26691

**Published:** 2022-07-09

**Authors:** Sumera Akram, Saeed Akhtar Khan Khattak, Muhammad A Khan

**Affiliations:** 1 Pediatrics and Neonatology, Fatima Jinnah Medical University, Lahore, PAK; 2 Pediatrics Department, District Headquarter Hospital (DHQ), Zhob, PAK; 3 Hematology, Pakistan Navy Station (PNS) Shifa Hospital, Karachi, PAK; 4 Otolaryngology - Head and Neck Surgery, National University of Medical Sciences, Rawalpindi, PAK

**Keywords:** transfusion dependent thalassemia, hydroxyurea, side effects, efficacy, beta thalassemia major

## Abstract

Background

Hydroxyurea is being used effectively in sickle cell anemia and thalassemia intermedia. Its role in transfusion-dependent beta-thalassemia major yet needs to be clearly established. This study has been carried out to assess the efficacy and safety of hydroxyurea as adjuvant therapy in pediatric cases of transfusion-dependent beta-thalassemia major disease.

Materials and methods

This quasi-experimental study was carried out at District Headquarter Hospital (DHQ), Zhob, from February 2021 to January 2022. One hundred ten cases fulfilling the inclusion-exclusion criteria were selected and divided into groups of 55 each. Group A cases received hydroxyurea (10-20 mg/kg/day) in addition to blood transfusion and chelation therapy. Group B received a blood transfusion and chelation therapy only. Both groups were compared in terms of blood transfusion requirement, mean hemoglobin, and mean serum ferritin levels. All the data were analyzed with SPSS 21 (IBM Corp., Armonk, NY).

Results

Of Group A cases, three were dropped because of side effects of hydroxyurea, and two were lost to follow-up. Similarly, three cases of Group B lost to follow-up, one patient withdrew consent, and one child died at home; thus both groups were left with 50 cases each. The mean age of participants was 11.98 + 3.74 years. There were 51 males and 49 females. Both the groups were comparable in terms of age and gender. Similarly, mean hemoglobin levels and serum ferritin levels were comparable at the start of the study. After one year, there was a significant improvement in mean hemoglobin level (p<0.001) and a significant reduction in serum ferritin levels (p=0.014) in the group taking adjuvant hydroxyurea. The requirement of packed red blood cells (RBCs) significantly decreased in cases taking hydroxyurea (p<0.001).

Conclusion

Hydroxyurea is a safe and effective treatment that significantly decreases the packed RBC transfusion requirement in transfusion-dependent thalassemia children, improves hemoglobin levels, and reduces serum ferritin levels compared to the children on blood transfusions alone.

## Introduction

Beta thalassemias are genetic diseases of hemoglobin synthesis where beta-globin subunits of hemoglobin are either absent or deficient [1]. Thalassemia has an autosomal recessive pattern of inheritance. Beta thalassemia has a high prevalence. Approximately 80 to 90 million people are reported to be carriers of this disease worldwide (1.5% of the global population) [2]. This enormously high frequency of hemoglobin disorder is because of natural selection mediated by the resistance of carriers against Plasmodium falciparum malaria and the high rate of consanguineous marriages in many countries [2]. These disorders are most prevalent in Southeastern and South Asia, the Middle East, the Mediterranean countries, and Africa [3].

The hemoglobin molecule is made up of two alpha and two beta chains. Beta thalassemia occurs due to reduced (beta+) or absent (beta zero) chains, with a relative excess of alpha chains [1]. The excess alpha-globin chains precipitate and cause oxidative damage to the cell membrane leading to ineffective erythropoiesis [4]. The clinical hallmark of the disease is anemia with peculiar signs and symptoms. The spectrum of the disorder varies from beta-thalassemia major to the beta-thalassemia intermedia and beta-thalassemia carrier state. The beta-thalassemia carrier state is clinically asymptomatic. Thalassemia major is the most severe form requiring frequent regular blood transfusion; also called transfusion-dependent thalassemia. Thalassemia intermedia is characterized by milder anemia, and such cases do not or occasionally require blood transfusions. Sometimes, individuals with thalassemia intermedia are asymptomatic until adult life [2,5].

Children with beta-thalassemia major require lifelong transfusion. Enhanced absorption of iron along with multiple recurrent transfusions puts them at risk of systemic iron overload. Iron deposits in the liver, heart, endocrine glands, and multiple other organs lead to multiple organ dysfunctions [1]. Modern iron chelators help restore iron overload in the body but the cost of managing becomes too high for the families and puts a financial burden on the health care system as well. In addition, chelating therapy requires strict compliance and monitoring of serum ferritin levels. The availability of iron chelators is also dubious in the areas of high prevalence. In poor and low-income countries with meager resources, the availability of safe blood is cumbersome and has an inherent risk of the transmission of life-threatening viral illnesses [6]. Many children in such countries die of transfusion-associated complications and iron overload, which is alarming. These complications include heart failure, arrhythmias, chronic hepatitis, chronic liver fibrosis/cirrhosis, etc [6-7].

Hemoglobin F is the main hemoglobin to carry oxygen during fetal life. After birth, hemoglobin F (HbF) is replaced by hemoglobin A (HbA), which becomes the predominant type [8]. The severity of thalassemia has been shown to be alleviated by HbF production, as it can alter alpha/beta-globin chain imbalance. It has also been linked with reduced morbidity and mortality in thalassemia cases and sickle cell disease patients [9].

Bone marrow transplantation is the only definitive cure at present. Modern therapies under study are fetal hemoglobin-inducing pharmacologic agents and stem cell therapy [4]. Hemoglobin F-inducing pharmacologic agents include hydroxyurea (hydroxycarbamide), 5-azacytidine, decytabine, and butyrate derivatives. Hydroxyurea has also been used with success in thalassemia patients with minimum side effects [2,6]. Hydroxyurea augments HbF reactivation of the gamma-globin gene, leading to gamma globin chain formation. These gamma chains combine with alpha chains to form HbF [6]. This drug has been used with success in thalassemia intermedia and sickle cell disease. Hydroxyurea is a low-cost medicine that results in a reduced need for transfusions in transfusion-dependent thalassemia [10-11]. It has been used in a dose of 10 mg/kg to 35 mg/kg as a single daily oral dose for thalassemia and sickle cell disease. The main side effects are liver and kidney dysfunction and bone marrow suppression [12].

District Zhob is one of the areas with the highest prevalence of thalassemia major in Pakistan [5]. Being a poor resource-constrained area with a lack of access to transport, difficulty in acquiring safe blood for transfusion regularly and dubious availability of very costly iron chelators made it a favorite area for conducting a study on the efficacy and safety of hydroxyurea in pediatric cases of thalassemia major.

## Materials and methods

This quasi-experimental study was carried out in District Headquarter Hospital, Zhob, from February 2021 to January 2022. Ethical permission was obtained from the hospital ethical board for the study (DHQ Hospital ERB, Approval number 32-21/P). Pediatric cases of beta-thalassemia major aged between five years and 18 years that were on regular blood transfusions and had consented to inclusion in this study were enrolled. Written consent was obtained from the parents of the patients for the study. Beta-thalassemia major cases with liver and renal dysfunction, massive splenomegaly, those with thrombocytopenia or leucopenia, or those who had any history of hypersensitivity to hydroxyurea were excluded.

A total of 110 cases fulfilling the inclusion-exclusion criteria were randomly selected from the thalassemia clinic of the hospital and enrolled. Out of these, 55 cases were prescribed hydroxyurea (10-20mg/kg/day) in addition to regular blood transfusion and chelation therapy and were labeled as Group A. The rest of the 55 cases continued receiving regular blood transfusions and chelation therapy and were labeled as Group B. Hydroxyurea was started initially at a dosage of 10 mg/kg/day as a single dose and gradually increased to 20 mg/kg/day over a period of one month. Both the groups were observed for one year and were compared in terms of transfusion requirement (number of packed red blood cells (RBCs) transfused per year), mean hemoglobin levels (before starting the study and after one year), and mean serum ferritin levels (at the start and after one year). All the cases were transfused group-specific, fully cross-matched, and compatible packed RBCs at 10 ml/kg. All the participants were prescribed deferasirox 20 mg/kg/dose daily as the chelating agent. Hemoglobin level was noted in grams/deciliters (gm/dL) and was measured two weeks after blood transfusion. Serum ferritin levels were measured in ng/dL. Serum alkaline transaminase (ALT) and serum creatinine were monitored monthly, if their value increased more than 1.5 times the normal value, hydroxyurea was stopped (considering it a side effect of hydroxyurea). Blood neutrophil count and platelet counts were investigated monthly. If the count of neutrophils decreased more than 1500/mm^3^ and platelet count decreased more than 100,000/mm^3^, hydroxyurea was withheld. The parents of participants were advised to report back if the children developed any side effects of hydroxyurea, i.e., headache, fever, nausea, vomiting, constipation, diarrhea, ulcers, mucositis, etc. A clinician would have stopped hydroxyurea if any child developed any of the above-mentioned side effects or neutropenia and thrombocytopenia [13].

The data, including age, gender, number of packed RBCs transfused per year, pre-transfusion hemoglobin levels at the start of the study and after one year, and serum ferritin levels before the start of the study and after one year, were entered and analyzed with Statistical Package for Social Sciences (SPSS 21; IBM Corp., Armonk, NY) and analyzed. Percentages were used to express frequencies, and chi-square was used to compare qualitative variables like gender and age groups and the independent t-test was applied for quantitative variables like age and hemoglobin levels. P-value < 0.05 was considered statistically significant.

## Results

Out of Group A cases, three were dropped because of the side effects of hydroxyurea and two were lost to follow-up. Similarly, three cases of Group B were lost to follow-up, one patient withdrew consent, and one child died at home; thus both groups were left with 50 cases each. The age range of participants was between five and 18 years and the mean age was 11.98 + 3.74 years. There were 51 males and 49 females among them as shown in Table 1.

**Table 1:** Demographic and Clinical Characteristics of Group A and Group B

S/No	Characteristic		Group A	Group B	Total	P-value
1	Age		12.08+3.66 years	11.88+3.84 years	11.98 + 3.74 years	0.790
2	Gender	Male	27	24	51	0.548
		Female	23	26	49	
3	Mean Hemoglobin at the Start of the Study		6.79+0.25 gm/dL	6.77+0.25 gm/dL	6.79 + 0.25 gm/dL	0.717
4	Mean Hemoglobin After One Year		8.33+0.53 gm/dL	7.04+0.41 gm/dL	7.68+0.80 gm/dL	0.000
5	Mean Ferritin Level at the Start of the Study		2237.26+719.88	2192.38+699.17	2214+706.37	0.752
6	Mean Ferritin Level After One Year		1680.98+542.44	1983.62+666.0	1832+623.14	0.014
7	Mean Transfusions per Year		9.62+1.44 transfusions/year	17.46+2.89 transfusions/year	13.54+4.55 transfusions/year	0.000

Both the groups were comparable in terms of age and gender. The mean age of Group A cases was 12.08+3.66 years and the mean age of Group B was 11.88+3.84 years. There were 27 males and 23 females in Group A and 24 males and 26 females in Group B. The mean hemoglobin level of Group A cases at the start of the study was 6.79+0.25 gm/dL and that of Group B was 6.77+0.25 gm/dL. The mean hemoglobin of both groups was comparable (p-value 0.717). After one year, the mean hemoglobin of Group A came to be 8.33+0.53 gm/dL versus 7.04+0.41 gm/dL in Group B. There was a significant difference between the hemoglobin levels of the two groups at the end of the study (p-value<0.001). The requirement of blood transfusions in Group A was significantly lower, i.e. 9.62+1.44 transfusions/year as compared to 17.46+2.89 transfusions/year in Group B (p-value<0.001). Similarly, mean serum ferritin also decreased significantly in Group A as compared to Group B (p-value being 0.014) as shown in Table 1.

## Discussion

In our study, we saw that hydroxyurea significantly improved the mean hemoglobin levels (p<0.001), significantly reduced the requirement of blood transfusions per year (p<0.000), and significantly decreased serum ferritin levels (p=0.014). There were three cases that experienced the side effects of hydroxyurea (two patients developed neutropenia and one had raised serum creatinine level; they recovered gradually after withdrawal of hydroxyurea).

Evidence has shown the clinical efficacy of hydroxyurea in patients with sickle cell disease and beta-thalassemia intermedia but there is a paucity of studies elaborating on the effectiveness and safety of hydroxyurea in transfusion-dependent beta-thalassemia major [6].

Sukhar K et al. enrolled 50 patients for studying the efficacy of hydroxyurea, however, 15 were dropped because of poor compliance (11), loss on follow-up (2), and side effects (2); forming the sample size of 35; however, they found a significant improvement in post-treatment hemoglobin levels [12]. Kosaryan M et al. studied 297 patients (248 cases of transfusion-dependent thalassemia major) and 48 cases of thalassemia intermedia); they claimed hydroxyurea to be very beneficial, as transfusions were completely stopped in 44.7% of transfusion-dependent thalassemia-major cases. Hydroxyurea was declared ineffective in 20% of cases and 13.4% of patients experienced the side effects of this therapy [14].

Yadav A et al. studied the efficacy of hydroxyurea in 25 cases of transfusion-dependent thalassemia-major. Hydroxyurea significantly increased mean hemoglobin levels and reduced the number of transfusions per day with no significant reportable side effects of therapy [15]. Ghosh d et al. studied the effect of hydroxyurea on 49 transfusion-dependent HbE/beta-thalassemia major cases and found significant improvement in the transfusion-free interval [16].

Bordbar MR et al. studied the effect of hydroxyurea therapy on 97 transfusion-dependent thalassemia-major cases and deduced that hydroxyurea significantly reduced the mean blood volume transfused and there was a significant improvement in post-treatment hemoglobin levels of patients with no serious side effects reported [17]. Yasara N et al. carried out a comprehensive review of the literature regarding the role of hydroxyurea in hemoglobinopathies. They have shown that hydroxyurea is an effective adjunct in transfusion-dependent thalassemia major, achieving a complete and partial response rate in >50% and 26% of participants, respectively [18].

The primary mechanism of action of hydroxyurea in beta hemoglobinopathies is the upregulation of gamma-globin gene expression in erythroid cells. The gamma-globin chains unite with alpha chains in red blood cells to synthesize HbF [18].

Hydroxyurea showed no serious side effects in almost all the studies conducted so far, except mild liver or renal impairment observed in a couple of researches [12-18]. It can be used safely in children as a single oral dose per day and monitored with complete blood count, alanine aminotransferase (ALT), and serum creatinine levels regularly. Withdrawal of this drug can reverse the biochemical alterations that occurred in a few studies [12,18].

The addition of hydroxyurea as an adjuvant in treating transfusion-dependent thalassemia-major cases will help reduce the blood transfusion requirement and its frequency, ameliorating the risk of transfusion-transmitted diseases and mitigating other complications associated with increased transfusion and iron overload, for instance, cardiac failure.

The limitation we came across in our study was measuring fetal hemoglobin (HbF) levels; that was not possible owing to resource constraints and the unavailability of this investigation in the whole area. We recommend randomized controlled trials with bigger/larger sample sizes and incorporating measurement of HbF levels as well.

## Conclusions

Hydroxyurea is a safe and effective treatment that significantly decreases the packed RBC transfusion requirement in transfusion-dependent thalassemia children, improves hemoglobin levels, and reduces serum ferritin levels compared to children on blood transfusions and iron chelators alone with a few mild side effects.
